# Ionized Hypomagnesemia Is Associated With Increased Incidence of Postoperative Atrial Fibrillation After Esophageal Resection: A Retrospective Study

**DOI:** 10.7759/cureus.17105

**Published:** 2021-08-11

**Authors:** Kotaro Hizuka, Takao Kato, Yuki Shiko, Yohei Kawasaki, Kaoru Koyama

**Affiliations:** 1 Anesthesiology, Saitama Medical Center, Saitama Medical University, Kawagoe, JPN; 2 Biostatistics, Clinical Research Center, Chiba University Hospital, Chiba, JPN; 3 Emergency Medicine, Japanese Red Cross College of Nursing, Tokyo, JPN

**Keywords:** postoperative atrial fibrillation, arrhythmia, thoracic surgery, esophageal cancer, electrolyte disturbance, ionized magnesium

## Abstract

Introduction: Postoperative atrial fibrillation (POAF) is common after surgery for esophageal cancer and may prolong hospitalization and elevate mortality. POAF and hypomagnesemia are linked, but this is based on studies showing an association of POAF with serum total magnesium (tMg). In contrast, the relationship of POAF with ionized magnesium (iMg), which has physiological activity, has not been examined. In this study, the association between hypomagnesemia and POAF was investigated retrospectively to examine iMg as a possible predictive factor for POAF.

Methods: The subjects were 151 patients who underwent right transthoracoabdominal subtotal esophagectomy at Saitama Medical Center between January 2011 and December 2020. The incidence of POAF and predictive factors were examined retrospectively. Perioperative predictive factors were subjected to univariate analysis, and items with P<0.1 were then included in multivariate analysis, along with five potential POAF predictors reported in the literature (age, gender, body mass index, hypertension, and diabetes mellitus). P<0.05 was regarded as significant in the multivariate analysis.

Results: Of the 151 patients, 34 (23%) developed POAF. In univariate analysis, six factors with P<0.1 (oral statin, dyslipidemia, iMg level after anesthesia induction, maximum and minimum iMg during surgery, and iMg level immediately before admission to ICU) were identified. In multivariate analysis including these and the five literature factors as explanatory variables, iMg immediately before admission to ICU emerged as a predictive factor for POAF (iMg≥0.46 mmol/L, OR 0.32, 95%CI 0.14-0.74, p=0.01) (standard iMg range: 0.48-0.60 mmol/L).

Conclusion: The iMg level immediately before admission to ICU may be associated with the development of POAF. A further study is needed to evaluate changes in iMg in the ICU and iMg at the time of onset of POAF.

## Introduction

Postoperative atrial fibrillation (POAF) occurs at a high rate and markedly increases the risk of cerebral and myocardial infarction, and associated mortality [1,2]. The causes of POAF include acute factors directly associated with surgery (inflammation and noradrenaline activation) and chronic or progressive factors, such as remodeling of the heart (left atrial enlargement), but the detailed pathophysiological mechanism has yet to be determined [3].

The incidence of POAF in surgeries other than cardiac or thoracic surgery is about 3% [4], whereas that after surgery for esophageal cancer is 16%, and this high rate is associated with an increased risk of postoperative complications, in addition to higher mortality [5]. The high incidence of POAF after surgery for esophageal cancer is due to the common patient background, which often includes cigarette smoking and alcohol intake, and to the physical stress of the operation on the heart, preoperative changes in atrial substrate, and noradrenaline stimulation due to surgical inflammation, all of which are thought to have effects because the esophagus is located close to the left atrium [3,6].

Studies of POAF after surgery for esophageal cancer have identified several preoperative predictive factors, including age, gender, body mass index (BMI), and a history of hypertension, diabetes mellitus, heart disease, or lung disease [2,3, 5,7-9]. None of these studies investigated intraoperative findings as predictive factors, but an association of intraoperative hypomagnesemia with POAF has been reported [10]. The Magnesium (Mg) level is generally measured as the serum total magnesium (tMg), but only ionized magnesium (iMg) has physiological activity and changes in iMg are inconsistent with changes in tMg [9]. The intraoperative iMg level has been found to be a risk factor for POAF after coronary artery bypass surgery using an artificial heart-lung machine [10].

In this study, we hypothesized that perioperative iMg is related to POAF after surgery for esophageal cancer. To examine this hypothesis, perioperative iMg was examined in patients undergoing right transthoracoabdominal surgery for esophageal cancer, and potential predictive factors for POAF were evaluated in an analysis of perioperative findings and previously identified patient-based risk factors.

## Materials and methods

A retrospective observational study was performed after approval by Saitama Medical Center Ethics Committee (approval number 2555-II). The STROBE checklist was utilized. The subjects were 185 patients who received curative surgery for esophageal cancer at Saitama Medical Center between January 1, 2011 and December 31, 2020, and were retrospectively followed for at least three months after surgery until March 31, 2021. Data were obtained from electronic medical records (EMRs). Patients who underwent elective right transthoracoabdominal subtotal esophagectomy were included in the study. Patients with atrial fibrillation (AF) induced by curative surgery for esophageal cancer using a different surgical procedure and those in whom AF was noted before surgery were excluded. Patients with no postoperative records available were also excluded from the study.

Surgery was performed under combined epidural and general anesthesia. General anesthesia was maintained by inhalation of sevoflurane or desflurane and total intravenous anesthesia with continuous administration of propofol, as selected by the physician in charge. For pain control, epidural anesthesia and remifentanil were concomitantly used during surgery and epidural anesthesia was used postoperatively. For differential lung thoracotomy, a double-lumen tube or bronchial blocker was used. After surgery, the patient was admitted to the ICU with intubation. Extubation was performed on the following day when there were no problems with consciousness, respiration, and circulation, and the patient was then managed in a general ward for two days after surgery.

The definition of POAF in this study was AF (including paroxysmal AF) that was newly developed within seven days after surgery. Continuous monitoring was used postoperatively in the ICU. After the patient returned to a general ward, arrhythmia was detected by a nurse during a routine vital signs check or a check for a symptomatic condition. Age, gender, BMI, history of hypertension or diabetes mellitus, and intraoperative iMg were obtained from EMRs.

Age, gender, height (cm), body weight (kg), BMI, resting heart rate/mean arterial pressure, the presence or absence of underlying disease (diabetes mellitus, dyslipidemia, hypertension, history of ischemic heart disease, cardiovascular disease, peripheral arterial disease), medications, cardiac ultrasonography findings (left atrial diameter, left ventricular ejection fraction), blood test results (hematocrit, potassium, creatinine, eGFR, ICGR15), ASA-PS classification, cancer stage, and the presence or absence of preoperative chemotherapy were examined as preoperative patient background factors. Operation time, fluid infusion/blood transfusion volume, blood loss, urine output, total balance, administered drugs, intraoperative body temperature, and perioperative blood test data (intraoperative iMg, potassium, blood glucose levels) were surveyed as perioperative factors. The presence or absence of POAF, hospitalization days and three months mortality were examined as postoperative outcomes. Intraoperative iMg was measured using a blood gas analyzer (Stat pHOx UltraTM, Nova Biomedical, Waltham, MA, USA). The standard value of iMg was set at 0.48-0.60 mmol/L [11].

Summary statistics (mean ± standard deviation) were calculated for continuous variables, and the number and rate of cases were calculated for categorical variables. To identify factors influencing the development of POAF, the subjects were divided into those with and without POAF. Univariate analysis of continuous variables was performed by Student t-test, and that of categorical variables was performed by Chi-squared test. Variables with P<0.1 in univariate analysis and five factors (age, gender, BMI, hypertension, and diabetes) associated with POAF in previous studies were used as explanatory variables in subsequent analyses. The cut-off value of each continuous variable was determined and the variable was binarized. Sensitivity and specificity were calculated using a ROC curve, and the Youden index was used as a reference. Multivariate logistic regression analysis using the stepwise method (threshold of P<0.1) was performed using the binarized factors as explanatory variables and the presence or absence of POAF as the response variable. When performing the stepwise method, the five variables (age, gender, BMI, hypertension, and diabetes mellitus) were fixed to remain in the analysis, and then the variable selection was performed for the other factors. A two-sided significance level of 5% was used in all statistical analyses. All calculations were performed in SAS ver. 9.4 (SAS Institute, Cary, NC, USA).

## Results

**Figure 1:**
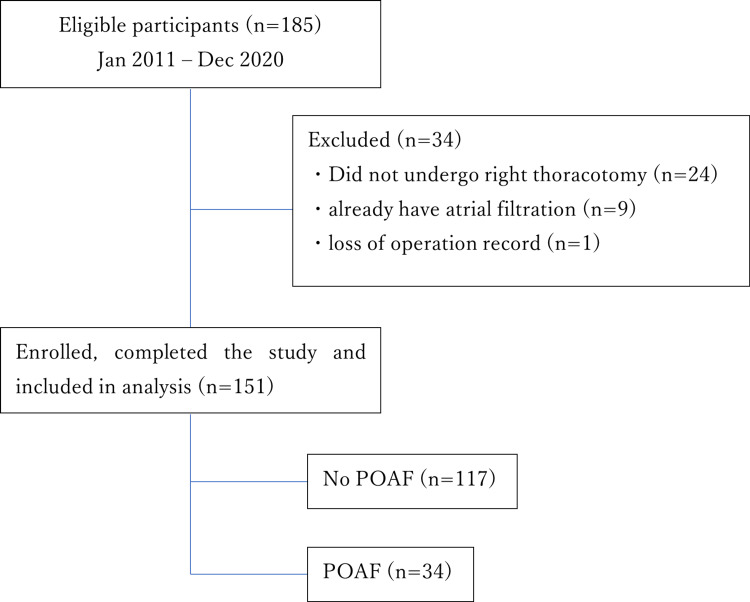
Flowchart for identifying patients between January 2011 and December 2020 at Saitama Medical Center, Saitama Medical University, Kawagoe, Japan POAF: postoperative atrial fibrillation

**Figure 2:**
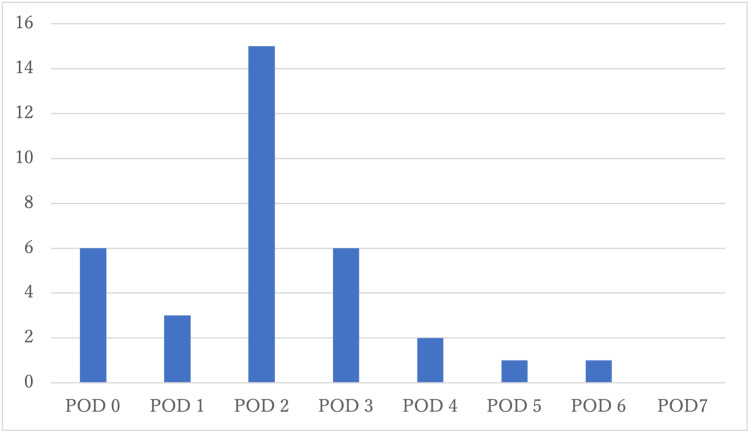
Onset of post-operative atrial fibrillation The most common onset of POAF was observed on the second postoperative day (15 cases, 44%). POD: postoperative day

Curative surgery for esophageal cancer was performed as an elective operation in 185 patients in total between January 2011 and December 2020, of whom 34 were excluded from the study. Of the remaining 151 patients, 34 developed POAF, giving an incidence of 23% (Figure 1). The POAF onset day and number of cases are shown in Figure 2. POAF most commonly developed two days after surgery in many cases (15 cases, 44%).

The results of univariate analysis are shown in Table 1 for preoperative factors and patient background, Table 2 for intraoperative factors, and Table 3 for postoperative factors. Table 1 shows oral statin administration and dyslipidemia were associated with a lower risk of POAF in preoperative factors. Table 2 shows iMg (before surgery, immediately before admission to ICU, maximum and minimum) were associated with a higher risk of POAF in intra-operative factors. Table 3 shows there were no significant differences in postoperative factors, including length of hospital stay and three months mortality. Due to the lack of iMg records in the ICU, we were unable to obtain data on postoperative iMg levels or on iMg levels at the onset of POAF. Oral statin administration, dyslipidemia, iMg level after anesthesia induction, intraoperative maximum and minimum iMg levels, and iMg level immediately before admission to ICU all had a value of P<0.1 in univariate analysis. Among these variables and the known factors, continuous variables were binary transformed for multivariate logistic regression analysis, and the respective cut-off values were calculated as follows, iMg immediately before admission to ICU: 0.46 mmol/L, iMg after induction of general anesthesia: 0.51 mmol/L, intra-operative Maximum iMg: 0.51 mmol/L, Intra-operative Minimum iMg: 0.46 mmol/L, Age: 72 years, BMI: 23 kg/m^2^ (Table 4). In this multivariate analysis, iMg immediately before admission to ICU emerged as an independent variable associated with POAF (≥0.46 mmol/L, OR:0.32, 95% CI: 0.14-0.74, p=0.01), in addition to the five previously reported factors (Table 5). Among the known factors, gender, hypertension, and diabetes mellitus did not show significant differences.

**Table 1:** Patient background (preoperative factors) and results of univariate analysis between no POAF and POAF patient Continuous data are presented as mean ± standard deviation and categorical data as number (percentage). POAF: post-operative atrial fibrillation; ICG-R15: Indocyanine green retention test after 15 minutes; ASA-PS: American Society of Anesthesiologists - Physical Status *eGFR was calculated by the equation devised by the Japanese Society of Nephrology [12].

	All patient (n=151)	No POAF (n=117)	POAF (n=34)	P-value
Age (years)	67.94±36.87	67.69±8.48	68.71±7.75	0.49
Gender (male)	131 (87%)	101 (86%)	30 (88%)	0.77
Height (cm)	162.83±7.61	162.48±7.81	164.03±6.89	0.30
Bodyweight (kg)	57.23±9.70	57.56±9.57	56.09±10.22	0.44
Body mass index (kg/m^2^)	21.57±3.32	21.79±3.22	20.82±3.60	0.14
Resting heart rate (bpm)	73.44±13.93	74.03±14.60	71.41±11.31	0.34
Resting mean arterial pressure (mmHg)	92.70±13.18	92.15±13.22	94.56±13.05	0.35
ICG-R15(%)	9.28±4.41	9.38±4.91	9.14±4.19	0.80
HbA1c (%)	5.99±0.83	6.01±0.91	5.88±0.67	0.52
Hematocrit (%)	37.52±4.83	37.38±4.89	38.01±4.65	0.50
Potassium (mEq/L)	4.39±0.418	4.39±.041	4.41±0.44	0.75
Cr (mg/dL)	0.84±0.26	0.85±0.29	0.80±0.14	0.34
eGFR* (ml/min/1.73 m^2^)	72.33±19.20	72.09±20.78	73.14±12.56	0.78
Left ventricular ejection fraction (%)	69.70±7.07	69.95±7.15	69.09±7.89	0.55
Left atrial diameter (mm)	33.66±5.55	33.58±5.88	34.12±5.55	0.64
Comorbidity				
Diabetes mellitus	16 (11%)	15 (13%)	1 (3%)	0.10
Dyslipidemia	19 (13%)	18 (15%)	1 (2.9%)	0.05
Hypertension	73 (48%)	60 (51%)	13 (38%)	0.18
Ischemic heart disease	6 (4%)	6 (5.1%)	0 (0%)	0.18
Cerebrovascular disease	12 (8%)	9 (7.7%)	3 (8.8%)	0.83
Peripheral arterial disease	4 (3%)	2 (1.7%)	2 (5.9%)	0.18
Medications				
Calcium blocker	53 (35%)	43 (37%)	10 (29%)	0.43
Angiotensin II receptor blocker or angiotensin-converting enzyme inhibitors	39 (26%)	30 (26%)	9 (26%)	0.92
beta-blocker	6 (4%)	5 (4.3%)	1 (2.9%)	0.73
alpha1-blocker	5 (3%)	5 (4.2%)	0 (0%)	0.22
Statin	14 (9%)	14 (12%)	0 (0%)	0.03
Aspirin	9 (6%)	6 (5.1%)	3 (8.8%)	0.42
Anticoagulant	10 (7%)	7 (6.0%)	3 (8.8%)	0.56
ASA-PS classification 1	29 (19%)	22 (19%)	7 (20%)	0.54
ASA-PS classification 2	118 (78%)	91 (78%)	27 (79%)	
ASA-PS classification 3	4 (3%)	4 (3%)	0 (0%)	
Cancer stage (0)	2(1%)	1(1%)	1(3%)	0.68
Cancer stage (Ⅰ)	15 (11%)	10(9%)	5(15%)	
Cancer stage (Ⅱ)	45 (30%)	36(31%)	9(26%)	
Cancer stage (Ⅲ)	77 (51%)	60(51%)	17(50%)	
Cancer stage (Ⅳ)	12 (8%)	10 (8.6%)	2 (6%)	
Preoperative chemotherapy	45 (30%)	38 (32%)	7 (21%)	0.18

**Table 2:** Intraoperative factors and results of univariate analysis between no POAF and POAF patients Continuous data are presented as mean ± standard deviation and categorical data as number (percentage). POAF: post-operative atrial fibrillation; iMg: ionized magnesium

	All patient (n=151)	No POAF (n=117)	POAF (n=34)	P-value
Potassium before surgery (mmol/L)	4.0±0.44	4.0±0.47	4.0±0.36	0.86
iMg before surgery (mmol/L)	0.52±0.07	0.53±0.07	0.49±0.07	0.0074
iMg immediately before admission to ICU (mmol/L)	0.49±0.08	0.51±0.08	0.45±0.07	0.0008
Maximum iMg (mmol/L)	0.54±0.08	0.55±0.09	0.51±0.06	0.0094
Minimum iMg (mmol/L)	0.47±0.07	0.48±0.08	0.44±0.07	0.023
Maximum–minimum iMg (mmol/L)	0.07±0.06	0.07±0.06	0.06±0.04	0.46
Blood glucose before surgery (mg/dl)	129±31	129±31	130±32	0.87
Maximum blood glucose (mg/dL)	189±40	189±43	188±31	0.94
Minimum body temperature (℃)	35±0.8	35±0.8	35±0.7	0.98
Body temperature after surgery (℃)	36±0.9	35±0.9	35±0.9	0.66
Magnesium sulfate	5 (3.3%)	5 (4.3%)	0 (%)	0.22
Insulin	18 (12%)	14 (12%)	4 (12%)	0.97
Noradrenaline	80 (52%)	66 (56%)	14 (41%)	0.12
Dopamine	11 (7.3%)	10 (8.6%)	1 (2.9%)	0.27
Dobutamine	4 (2.6%)	2 (1.7%)	2 (5.9%)	0.18
Operation time (min)	393±78	391±75	403±87	0.43
Total fluid infusion (mL)	3,950±1,197	3,963±1,183	3,904±1258	0.80
Blood transfusion (red blood cell, mL)	177±368	175±379	181±330	0.93
Blood transfusion (fresh frozen plasma, mL)	78±223	78±220	78±234	0.99
Blood loss (mL)	680±587	663±524	736±771	0.52
Urine output (mL)	1,063±700	1,107±733	910±557	0.15
Total balance (mL)	2,462±1,019	2,447±994	2,517±1,115	0.73

**Table 3:** Postoperative factors and results of univariate analysis between no POAF and POAF patients Continuous data are presented as mean ± standard deviation and categorical data as number (percentage). POAF: post-operative atrial fibrillation; POD: postoperative day *The Kidney Disease Improving Global Outcomes (KDIGO) classification was used for the diagnosis of acute kidney injury.

	All patient (n=151)	No POAF (n=117)	POAF (n=34)	P-value
Noradrenaline	40 (26%)	30 (26%)	10 (29%)	0.66
C-reactive protein (mg/dL)	13±5.5	13±5.3	13±6.1	0.79
Creatinine kinase (U/L)	823±375	803±402	909±456	0.23
Acute kidney injury*	4 (2.6%)	2 (1.7%)	2 (5.9%)	0.18
Extubation (POD)	1.2±1.1	1.1±1.1	1.4±1.1	0.24
Hospitalization days	45±37	56±54	42±31	0.15
Three months mortality	2 (1.3%)	1 (0.9%)	1 (2.9%)	0.35

**Table 4:** Binary transformed the analysis of continuous variables of candidate predictors Data are presented as numbers (percentage). POAF: postoperative atrial fibrillation; iMg: ionized magnesium; ICU: intensive care unit

		No POAF		POAF		P-value
		Number	%	Number	%	
iMg immediately before admission to ICU（mmol/L）	<0.46	28	24	18	53	0.0012
	≥0.46	89	76	16	47	
iMg after induction of general anesthesia (mmol/L)	<0.51	40	34	21	62	0.0039
	≥0.51	77	66	13	38	
Intra-operative maximum iMg (mmol/L)	<0.51	34	29	19	56	0.0039
	≥0.51	83	71	15	44	
Intra-operative minimum iMg (mmol/L)	<0.46	44	38	19	56	0.057
	≥0.46	73	62	15	44	
Age（years）	<72	76	65	16	47	0.060
	≥72	41	35	18	53	
Body mass index（kg/m^2^）	<23	72	62	28	82	0.024
	≥23	45	38	6	18	

**Table 5:** Predictors of postoperative atrial fibrillation by multivariate analysis iMg: ionized magnesium; ICU: intensive care unit

Variables		Odds Ratio	95% Confidence Interval		P-value
Age (years)	≥72	2.86	1.19	6.86	0.02
Gender	Male	1.77	0.48	6.51	0.39
Body mass index (kg/m^2^)	≥23	0.28	0.10	0.80	0.02
Hypertension	No morbidity	1.80	0.74	4.35	0.19
Diabetes mellitus	No morbidity	5.00	0.56	44.36	0.15
iMg immediately before admission to ICU（mmol/L）	≥0.46	0.32	0.14	0.74	0.01

## Discussion

In this study, 23% of patients developed POAF after elective right transthoracoabdominal subtotal esophagectomy. In multivariate analysis, iMg immediately before admission to ICU was identified as an independent POAF-predictive factor. POAF most frequently developed two days after surgery and 34% of all cases occurred within two days after surgery.

Hypomagnesemia (tMg <1.5 mg/dl or <0.62 mmol/L) is common in patients with advanced cancer and in those admitted to an ICU [13,14]. Cisplatin used in preoperative chemotherapy for esophageal cancer induces hypomagnesemia [15], and thus, patients undergoing surgery for esophageal cancer are more likely to have hypomagnesemia compared with other cases. The action potential of cardiac muscle cells is transmitted through voltage-dependent sodium, potassium, and calcium ion channels, and changes in the function of these channels may lead to arrhythmia. Mg acts as a membrane‐stabilizer by blocking calcium channels and adjusting cell membrane sodium-potassium transport and also inhibits catecholamine release from the adrenal gland and adrenergic nerve ending [16]. These properties result in an anti-arrhythmic effect of Mg. Similar to esophageal cancer surgery, POAF is common after coronary artery bypass surgery, in which an association of hypomagnesemia and prevention of POAF by magnesium preparations have been shown [17-19]. However, tMg was examined in these studies and iMg was rarely measured, despite its association with physiological activity [20]. Also, changes in iMg are inconsistent with changes in tMg, and tMg may be within the normal range despite the intracellular magnesium content being low [9,21].

The current study showed the value of measuring iMg as a direct predictor of POAF. The cut-off value of iMg, 0.46 mmol/L, was consistent with the range of clinical hypo-ionized hypomagnesemia [11], suggesting an indication for magnesium sulfate supplementation. Magnesium supplementation targeting iMg ≥0.50 mmol/L during artificial heart-lung management has been shown to decrease the frequency of ventricular tachycardia and prolong the duration of sinus rhythm in coronary artery bypass surgery [22]. Setting a target value of iMg, which can be easily measured at the bedside, has been found to facilitate favorable management in the treatment of Torsades de pointes accompanying drug-induced long QT syndrome [23]. Magnesium supplementation has also been suggested to be useful for the prevention of POAF after surgery for esophageal cancer and cardiac surgery [6,24,25], and thus, perioperative iMg monitoring and Mg supplementation may prevent POAF.

The basis of the development of POAF after surgery for esophageal cancer is not completely clear, but the mechanism is multifactorial and various predictive factors have been investigated. In this study, the multivariate analysis included five previously established risk factors (age, gender, BMI, hypertension, and diabetes mellitus). In the 2014 AATS Guidelines for Prevention and Management of Perioperative Atrial Fibrillation and Flutter for Thoracic Surgical Procedures [6], age, gender, BMI, and hypertension are included as risk factors. Among these predictive factors, the most consistent is advanced age, and aging-associated atrial tissue remodeling is thought to increase the risk of POAF. Hypertension and diabetes mellitus can also alter atrial tissue [26]. Perioperative statin administration has been reported to decrease the incidence of POAF [27], and in the current study, the incidence of POAF was significantly lower in patients taking oral statins for hyperlipidemia in univariate analysis.

POAF developed after surgery for esophageal cancer in 34 (23%) of the 151 patients in the current study. The most frequent onset time was two days after surgery, which occurred in 44% of cases, and POAF developed three days after surgery in 88% of the affected cases. In previous studies of esophageal cancer surgery, the incidence of POAF was ≥15% and the findings were consistent with our observation of peak development two days after surgery [4,6]. Few studies have examined the association of iMg with physiological activity and POAF development [20] and this is the first report to examine the relationship of iMg with POAF development after surgery for esophageal cancer.

This study has several limitations, including its retrospective design. A larger number of cases could not be included because medical records were not kept for more than 10 years, and this reduced our ability to adjust for confounding factors. As a result, of the factors identified in previous studies, there was no significant difference in gender, hypertension, or diabetes mellitus between POAF and non-POAF cases. Also, outcomes, such as mortality, could not be investigated because the number of cases was small and the incidence was low. In addition, tMg was not measured, which prevented examination of the correlation of tMg with POAF. There was no record of Mg measurement in the postoperative period, including in the ICU, so we were unable to investigate the relationship between iMg at the time of onset of POAF and that during POAF.

## Conclusions

Predictive factors for POAF after surgery for esophageal cancer were investigated in a single-center retrospective study. In multivariate analysis, iMg immediately before admission to ICU was identified as a risk factor for POAF (iMg≥0.46 mmol/L, OR:0.32, 95% CI: 0.14-0.74, p=0.01). This is the first study to show a possible association between ionized magnesium and POAF after esophageal resection. Further prospective studies on the relationship between ionized magnesium and POAF (and furthermore, the relationship with mortality) are needed.
